# Medial Subluxation or Dislocation of the Biceps on Magnetic Resonance Arthrography Is Reliably Correlated with Concurrent Subscapularis Full-Thickness Tears Confirmed Arthroscopically

**DOI:** 10.1155/2018/2674061

**Published:** 2018-09-09

**Authors:** Ji-Sang Yoon, Sung-Jae Kim, Yun-Rak Choi, Wonyong Lee, Sang Ho Kim, Yong-Min Chun

**Affiliations:** The Department of Orthopaedic Surgery, Arthroscopy and Joint Research Institute, Severance Hospital, Yonsei University College of Medicine, Seoul, Republic of Korea

## Abstract

**Background:**

The purpose of this study was to investigate the relationship between biceps medial subluxation/dislocation on the magnetic resonance arthrography (MRA) imaging and subscapularis full-thickness tear confirmed arthroscopically. We hypothesized that presence of a biceps medial subluxation or dislocation would strongly indicate a subscapularis full-thickness tear.

**Methods:**

A total of 432 consecutive patients who underwent arthroscopic repair for rotator cuff tears with/without subscapularis tears at our institute were retrospectively reviewed. The inclusion criterion of this study was preoperative MRA images taken within 6 months of arthroscopic repair. The presence of medial subluxation/dislocation was evaluated on the preoperative MRA images, and subscapularis tear was confirmed on arthroscopic examination.

**Results:**

Biceps subluxation/dislocation was identified in 46 of the 432 patients on MRA. Forty-five of these 46 patients also had a subscapularis full-thickness tear identified in arthroscopic examination. Among the 386 patients who did not have biceps subluxation or dislocation, 54 patients had a subscapularis full-thickness tear diagnosed arthroscopically. The presence of a biceps subluxation/dislocation could predict a subscapularis full-thickness tear with sensitivity of 45% (45/99), specificity of 99% (332/333), positive predictive value of 98% (45/46), negative predictive value of 86% (332/386), and accuracy of 87% (377(45 +332)/432).

**Conclusion:**

Medial subluxation/dislocation of the biceps on MRA images was highly associated with a concurrent subscapularis full-thickness tear which was confirmed arthroscopically. This association had 99% specificity and 98% positive predictive value. Therefore, if a biceps subluxation/dislocation is identified on MRA images, there is a high chance that a concurrent subscapularis full-thickness tear exists.

## 1. Introduction

Various imaging modalities can be used to detect rotator cuff tears. In particular, magnetic resonance imaging (MRI) is an effective tool with high sensitivity and specificity for diagnosing supraspinatus and infraspinatus tears. However, several studies have indicated that MRI and even magnetic resonance arthrography (MRA) do not reliably predict subscapularis tears [1–4]. There are several physical examinations used to detect subscapularis tears, including the lift-off test, belly press test, and bear hug test; however, these maneuvers have relatively low sensitivity [5, 6]. Unfortunately, many subscapularis tears may be overlooked clinically.

**Figure 1:**
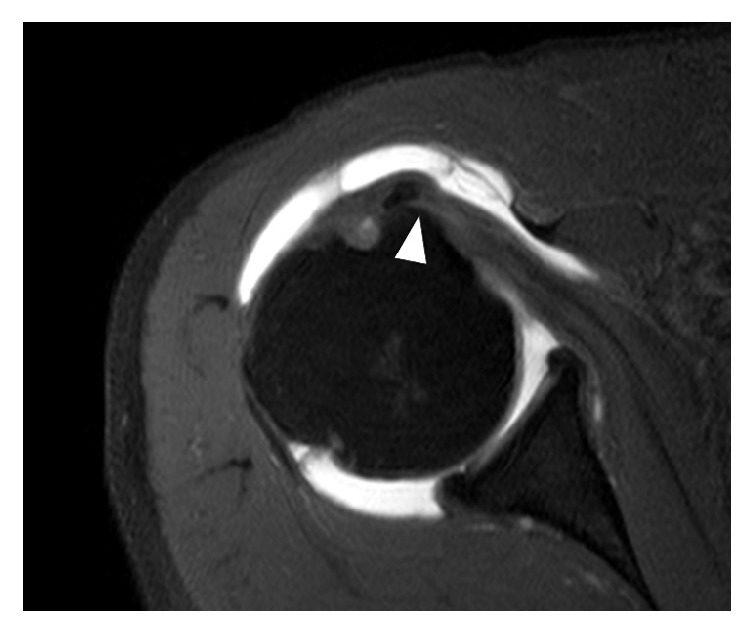
A biceps medial dislocation protrudes out of bicipital groove on an axial oblique image of MR arthrography in the right shoulder. Although the insertion of the subscapularis tendon on the footprint seems to be intact, a full-thickness tear at the upper portion of the subscapularis tendon was identified arthroscopically. There is increased signal intensity (arrow head) at the tear site.

At an early stage of a subscapularis full-thickness tear without retraction, it is often difficult to differentiate between a normal insertion and tear. This is particularly true if the tear is limited to the upper portion of the tendon. If the MR image does not exactly capture the torn part of the tendon, it may appear to attach well to the lesser tuberosity. Thorough and meticulous investigation of MR images may identify a subtle change in signal intensity (Figure 1).

The superolateral border of the subscapularis tendon is connected to the biceps pulley, which precludes medial displacement of the biceps. Considering that subscapularis tears are likely to involve the biceps pulley and lead to medial displacement of the biceps subsequently, a biceps subluxation or dislocation can be a reliable clue of an underlying subscapularis tear [1, 2, 7–9].

The purpose of this study was to investigate the relationship between biceps medial subluxation/dislocation on the MRA images and subscapularis full-thickness tear which was confirmed arthroscopically. We hypothesized that presence of the biceps medial subluxation/dislocation would strongly indicate a concurrent subscapularis full-thickness tear.

## 2. Materials and Methods

**Figure 2:**
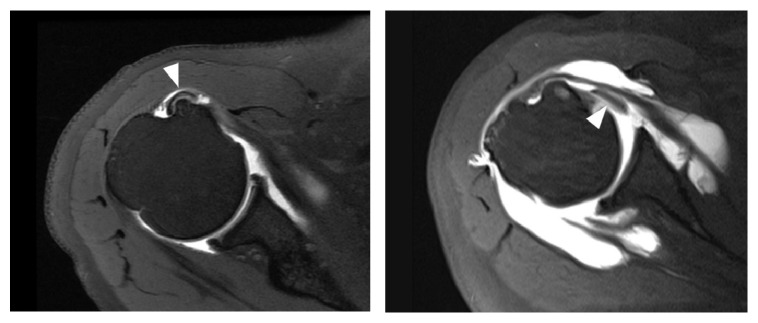
(a) A biceps medial subluxation (white arrow head) over the bicipital groove and lesser tuberosity, right shoulder. (b) A biceps medial dislocation (white arrow head) in the setting of a subscapularis full-thickness tear, right shoulder.

We retrospectively reviewed a consecutive series of patients who underwent arthroscopic repair for rotator cuff tears either with or without subscapularis full-thickness tears at our institute between January 2010 and August 2015. The inclusion criterion of this study was preoperative MR arthrography (MRA) (3.0T MR imager, Magnetom Tim Trio, Siemens, Erlangen, Germany) taken in neutral position of the affected shoulder at our institute, within 6 months prior to arthroscopic repair [1, 7]. The exclusion criteria were MRA images obtained at other institutes and history of previous shoulder surgery. Biceps subluxation and dislocation were defined on axial images as biceps displacement over the lesser tuberosity and bicipital groove (subluxation) (Figure 2(a)) or completely out of the bicipital groove (dislocation) (Figure 2(b)). An experienced shoulder specialist (one of senior authors) who was blinded to arthroscopic findings reviewed MRA images of the included patients and determined the presence of the biceps subluxation/dislocation on the axial images. This study was approved by our institutional review board, and the need for informed consent was waived.

**Figure 3:**
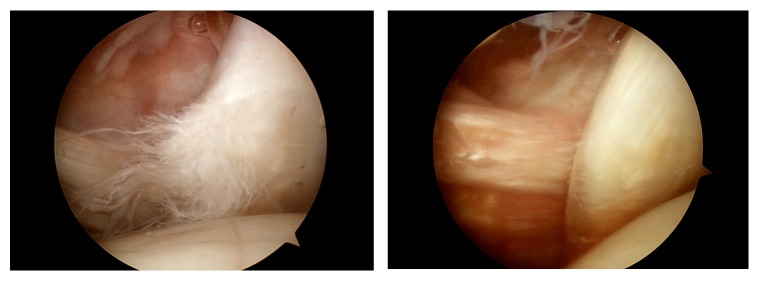
(a) Biceps medial subluxation. The biceps is located behind the torn subscapularis tendon, viewed from the posterior portal, right shoulder. (b) Biceps medial dislocation. The biceps is placed behind the torn subscapularis tendon and completely out of the bicipital groove, viewed from the posterior portal, right shoulder.

Arthroscopic evaluation was performed by a single surgeon in a beach-chair position under general anesthesia. Both 30° and 70° arthroscopes were used to investigate the articular and bursal side of the subscapularis insertion. With use of the 70° arthroscope, as indicated by several studies, the footprint of the subscapularis as well as tear within the bicipital groove can be more clearly visualized [10–12]. Through the standard posterior portal, the footprint of the subscapularis tendon, location of the biceps, their relation to the biceps pulley, and structural integrity of the biceps pulley itself were investigated (Figure 3).

We defined the full-thickness tear of the subscapularis as complete detachment of the subscapularis from its footprint on the lesser tuberosity, which was confirmed under arthroscopic examination. Despite a full-thickness tear, in many cases, the torn edge of the subscapularis tendon was connected to the transverse ligament over the bicipital groove and, subsequently, the torn subscapularis tendon remained in place without any medial retraction. These were also regarded as full-thickness tears.

## 3. Results

This study included 432 patients (189 male and 243 female), and their mean age at the time of surgery was 63.2 years (range, 47 to 80 years). Among them, biceps subluxation/dislocation was identified in 46 patients on preoperative MRA (intraclass correlation coefficient of intraobserver reliability = 0.857). Of these 46 patients, 45 patients also had a subscapularis full-thickness tear identified on arthroscopic examination. Of 386 patients who did not have biceps subluxation/dislocation on preoperative MRA, 54 patients had subscapularis full-thickness tears diagnosed arthroscopically (Table 1). The presence of a biceps subluxation/dislocation could predict the subscapularis full-thickness tear with sensitivity of 45% (45/99), specificity of 99% (332/333), positive predictive value of 98% (45/46), negative predictive value of 86% (332/386), and accuracy of 87% (377(45 +332)/432).

## 4. Discussion

Subscapularis tears seem to be slightly different than supraspinatus tears. A supraspinatus full-thickness tear can be easily identified because, regardless of the degree of retraction, complete detachment of the cuff from its footprint is detected on MR imaging. However, even in full-thickness subscapularis tears, the subscapularis tendon often appears to attach to its footprint in the setting of biceps subluxation/dislocation. This may be attributed to the transverse ligament over the bicipital groove. The transverse ligament seems to hold the subscapularis tendon in place, preventing it from retracting medially even after a full-thickness tear [13]. Therefore, unlike other supraspinatus or infraspinatus tears, it is often difficult to clearly identify subscapularis tears on the MR images. Consistent with our original hypothesis, biceps subluxation/dislocation was highly associated with concurrent subscapularis full-thickness tear. This association has both a high positive predictive value and specificity. On the other hand, there were also many subscapularis full-thickness tears in the absence of biceps subluxation/dislocation. Therefore, the sensitivity of this association was low in this study. Nevertheless, the negative predictive value and accuracy were relatively high.

Interestingly, Shi et al. recently reported that the diagnostic value of biceps subluxation lies in the negative predictive value; if there is no biceps subluxation, then it is unlikely that there is a subscapularis full-thickness tear [7]. In contrast to the current study, the group reported a positive predictive value of 35% and negative predictive value of 97%; among 26 patients diagnosed with biceps long head subluxation, only 9 patients had full-thickness subscapularis tears confirmed arthroscopically. In our data, biceps subluxation/dislocation in the absence of subscapularis full-thickness tear was only one case and it seems to be rare, although there are previous case reports that describe biceps dislocation without rotator cuff tears [14, 15]. Nevertheless, the incidence of biceps subluxation without subscapularis tear in their study was relatively high. Based on their study and current study, the accuracy itself for the diagnosis of the biceps subluxation or dislocation based on MR axial image seems to be important for predicting the subscapularis full-thickness tear. In the current study, its accuracy was 96.7 % when employing the 3.0 T MRA, although direct comparison was not feasible because there was no comment on this issue in other studies.

Lafosse et al. used arthroscopic static and dynamic evaluation to classify biceps instability in rotator cuff tears as anterior or posterior instability [16]. In their study, anterior instability of the biceps tendon, which is consistent with current biceps subluxation/dislocation over the bicipital groove, was often related to a subscapularis tear. In contrast, posterior instability was more related to a supraspinatus tear. Among 200 consecutive cases, anterior and anteroposterior biceps instability were identified in as many as 26% of cases, which is a higher rate than that in the current study, and in others [7, 9, 17]. This finding is attributable to the fact that our study and the others mentioned exclusively addressed anterior static instability and not posterior or dynamic instability.

**Figure 4:**
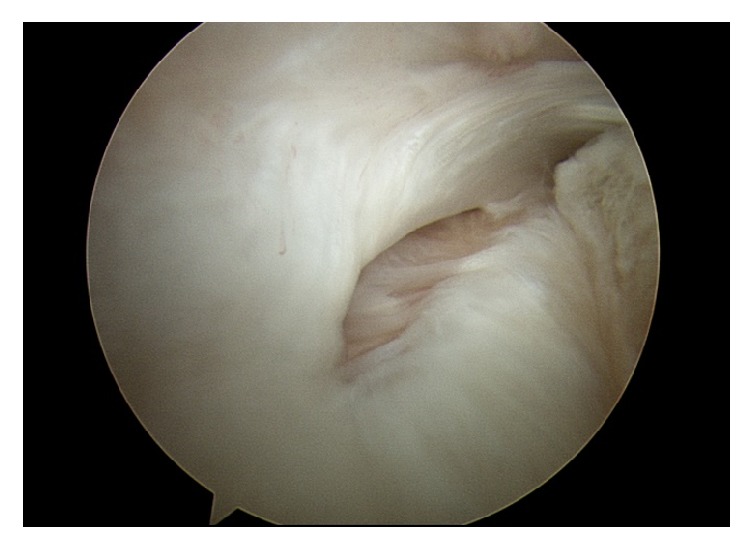
A subscapularis interstitial tear identified within the bicipital groove, viewed from the posterior portal using a 70° arthroscope, right shoulder.

Walch et al. reported several types of biceps subluxations/dislocations that were associated with the status of the adjacent subscapularis tear [9]. Among the various types, there was a biceps dislocation inside a subscapularis tear that resulted in an interstitial subscapularis partial-thickness tear. In the current study, there were similar cases, although there was no biceps subluxation/dislocation; the tear existed within the bicipital groove, which could be only identified using a 70° arthroscope into the bicipital groove (Figure 4). It was very difficult to identify this lesion on preoperative MR image, including MRA. In addition, there were no instances of biceps subluxation/dislocation in interstitial subscapularis partial-thickness tears in the current study.

**Figure 5:**
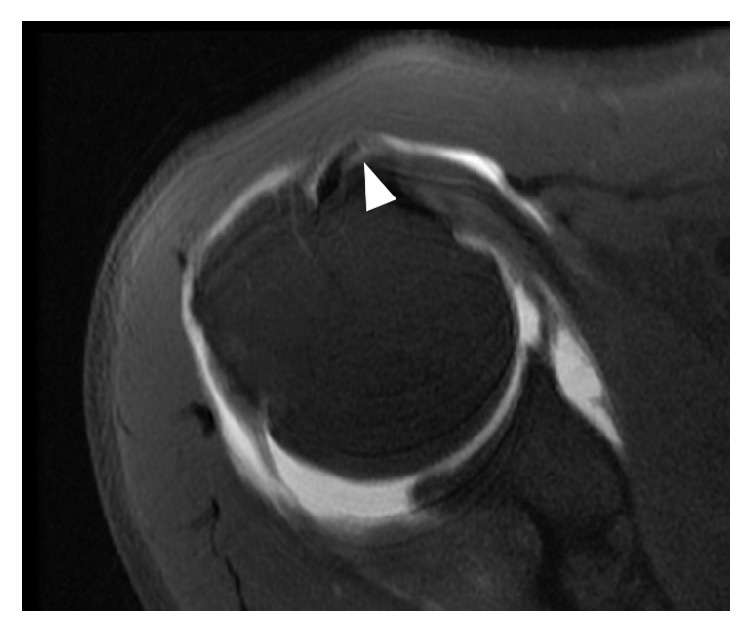
A biceps medial subluxation without a subscapularis full-thickness tear, right shoulder. A subscapularis partial-thickness tear (white arrow head) within the bicipital groove was identified.

The vast majority (90%) of subscapularis tears started from the articular side and cephalad portion of the tendon [1, 2, 18, 19]. Therefore, biceps medial subluxation/dislocation appears to be a result of subscapularis full-thickness tears, even though interstitial or bursal side partial-thickness subscapularis tears can exist. It is likely that the subscapularis tear starts from the articular side as a partial-thickness tear and extends toward the lateral side. Finally, the tear may disrupt the medial sling of the biceps, connected with the bicipital groove. Eventually, the biceps can be medially subluxated or dislocated through an unstable biceps sling. Therefore, when biceps medial subluxation/dislocation is identified, causative subscapularis full-thickness tear seems to be an inevitable consequence. In the current study, there was only one case of biceps subluxation diagnosed on preoperative MR image without a concomitant subscapularis full-thickness tear (Figure 5). Arthroscopic examination revealed subscapularis partial-thickness tear within the bicipital groove with an intact articular portion of the subscapularis tendon.

This study has several limitations. First, this study evaluated just static anterior biceps instability, rather than assessing a dynamic instability depending on the position or rotation of the shoulder. Our results might be strengthened by a dynamic study of biceps instability using ultrasonography. Second, one senior author determined the presence of the biceps subluxation/dislocation on the MRA axial image. Third, we limited 3.0 T MRA image evaluated in our institute to get the uniformity and accuracy of the images. However, in contrast, in a clinical setting, it might have been more realistic to include MR images from various sources.

## 5. Conclusion

Medial subluxation or dislocation of the biceps on MRA images was highly associated with a concurrent subscapularis full-thickness tear confirmed arthroscopically. This association had 99% specificity and 98% positive predictive value. Therefore, if a biceps subluxation/dislocation is identified on MRA images, there is a high chance that a concurrent subscapularis full-thickness tear exists.

## Figures and Tables

**Table 1:** Biceps subluxation/dislocation and subscapularis tear.

	Subscapularis full-thickness tear	SSc partial-thickness tear or no tear	total
Subluxation/Dislocation (+)	45	1	46

Subluxation/Dislocation (-)	54	332	386

Total	99	333	432

## Data Availability

The data used to support the findings of this study are available from the corresponding author upon request.
